# Nanoparticles in Medicine: A Focus on Vascular Oxidative Stress

**DOI:** 10.1155/2018/6231482

**Published:** 2018-09-26

**Authors:** M. D. Mauricio, S. Guerra-Ojeda, P. Marchio, S. L. Valles, M. Aldasoro, I. Escribano-Lopez, J. R. Herance, M. Rocha, J. M. Vila, V. M. Victor

**Affiliations:** ^1^Department of Physiology, Faculty of Medicine and Odontology, Universitat de Valencia and Institute of Health Research INCLIVA, Valencia, Spain; ^2^Service of Endocrinology, University Hospital Doctor Peset, Foundation for the Promotion of Health and Biomedical Research in the Valencian Region (FISABIO), Valencia, Spain; ^3^Medical Molecular Imaging Research Group, Vall d'Hebron Research Institute (VHIR), CIBBIM Nanomedicine, Passeig de la Vall d'Hebron, Barcelona, Spain

## Abstract

Nanotechnology has had a significant impact on medicine in recent years, its application being referred to as nanomedicine. Nanoparticles have certain properties with biomedical applications; however, in some situations, they have demonstrated cell toxicity, which has caused concern surrounding their clinical use. In this review, we focus on two aspects: first, we summarize the types of nanoparticles according to their chemical composition and the general characteristics of their use in medicine, and second, we review the applications of nanoparticles in vascular alteration, especially in endothelial dysfunction related to oxidative stress. This condition can lead to a reduction in nitric oxide (NO) bioavailability, consequently affecting vascular tone regulation and endothelial dysfunction, which is the first phase in the development of cardiovascular diseases. Therefore, nanoparticles with antioxidant properties may improve vascular dysfunction associated with hypertension, diabetes mellitus, or atherosclerosis.

## 1. Introduction

The emergence of nanotechnology and its convergence with other disciplines such as biomaterial science, cell and molecular biology, and medicine, referred to as nanomedicine, have drawn the attention of biomedical research due to its potential applications in the diagnosis and treatment of diseases. Nanoparticles (NPs) are the main system used in nanomedicine, as theranostic agents with high molecular specificity [1–3]. Due to their size (1–100 nm), nanoparticles have a large surface area-to-volume ratio, which allows them to absorb high quantities of drugs [4] and to be spread easily throughout the bloodstream [5]. Their larger surface area gives them unique characteristics, as it improves their mechanical, magnetic, optical, and catalytic properties, thus increasing their potential pharmacological use [4].

Studies on the potential effects and benefits of NPs in diseases involving oxidative stress are receiving growing attention. Cardiovascular risk factors such as hypercholesterolemia or hypertension promote the generation of reactive oxygen species (ROS), which leads to the oxidative stress seen in inflammatory diseases such as atherosclerosis [6]. Therefore, the maintenance and optimization of antioxidant defences can minimize side effects. In this sense, nanoparticles are of great interest, because of their antioxidant properties and easy internalization by the cells.

In this review, we discuss the main mechanisms of oxidative stress implicated in the development and progression of vascular diseases. We also summarize current knowledge in relation to each pathway and mention some examples of the use of NPs as theranostic agents.

## 2. Nanoparticles in Medicine

### 2.1. General Characteristics

The rapid development of nanotechnology for biological purposes has had a tremendous impact on medicine. Nanotechnology enables the manufacture and manipulation of materials on a nanometer scale, thus allowing the development of new tools for the treatment, diagnosis, monitoring, and control of biological systems. This application of nanotechnology in the field of medicine is known as nanomedicine. Nanoparticles, the most widely used nanotechnology platforms in nanomedicine, are particles with two or more dimensions on the nanometer scale, according to the American Society for Testing and Materials (ASTM). These NPs have special enhanced physical and chemical properties compared to their corresponding bulk materials. These properties include a high surface area-to-volume ratio and a unique quantum size effect due to specific electronic structures [7]. In addition to their composition, the properties of NPs depend on their size and shape [8]. Generally, in order to obtain monodispersed NPs and facilitate their internalization by cells, it is necessary to control their size and shape and thus minimize aggregation [9]. It is because of these properties that NPs have been considered as diagnostic, therapeutic, and carrier agents in biomedical applications [1–3]. For instance, some applications are thanked for their surface-mass ratio, which is greater than that of other particles and enables them to bind to, absorb, or carry other molecules [3]. Furthermore, they can be modified or manufactured with two or more materials to improve their physical properties.

### 2.2. Types of Nanoparticles

**Figure 1:**
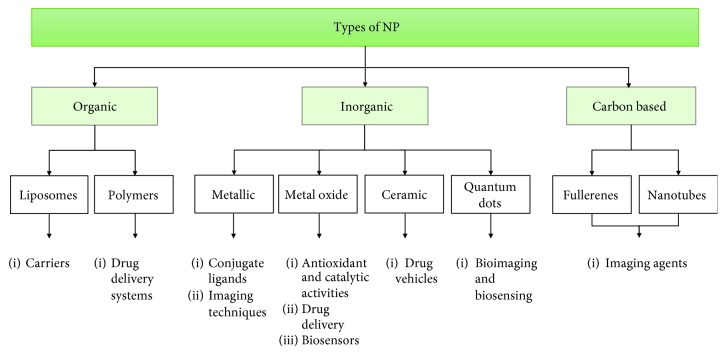
Generalized diagram of the types of nanoparticles and their main biomedical applications. Based on their chemical composition, nanoparticles can be divided into three main groups: organic, inorganic, and carbon-based. Each category includes several types of nanoformulations.

Regarding their chemical compounds, NPs can be divided into three main groups: organic nanoparticles (liposomes and polymers), inorganic nanoparticles (metals, metal oxide, ceramic, and quantum dots), and carbon-based nanoparticles [10] (Figure 1). In general, NPs retain the chemical properties of their bulk materials, which can be useful when choosing a specific NP for a biomedical application. The NPs used in nanomedicine include the following.

#### 2.2.1. Liposome Nanoparticles

These are spherical vesicles with a membrane composed of a lipid bilayer containing an aqueous substance. The amphiphilic molecules used for the preparation of these vesicles are similar to biological membranes so as to improve the efficacy and safety of different drugs [11]. The active compound can be hydrophilic and therefore located in the aqueous space or hydrophobic, remaining in the lipid membrane. The synthesis of a liposome depends mainly on the following parameters: (a) the physicochemical characteristics of the material to be entrapped and those of the liposomal compounds; (b) the nature of the medium in which the lipid vesicles are dissolved, the concentration of the entrapped substance, and its potential toxicity; (c) additional processes implicated in during the fabrication, application, or delivery of the vesicles; (d) dispersity, size, and shelf-life of the vesicles for the intended application; and (e) batch-to-batch reproducibility and possibility of large-scale production of safe and efficient liposomal products. Liposomes can be synthetized by sonicating a dispersion of amphipatic lipids, such as phospholipids, in water. In fact, low shear rates can create multilamellar liposomes. The original aggregates have many layers, thus forming progressively smaller, eventually unilamellar liposomes. Sonication is a “gross” method of preparation, as it can damage the structure of the drug to be encapsulated. In addition, there are other methods, such as extrusion and the Mozafari method [12], which are employed to produce materials for human use. Finally, it is important to mention that using lipids other than phosphatidylcholine can greatly facilitate liposome preparation.

Liposomes are mainly used for delivering chemotherapeutic drugs in cancer treatment [13]. They can also incorporate a high number of bioactive materials, including pharmaceutical drugs or food ingredients. Liposomes have great potential applications in nanomedicine, as well as in the food and cosmetics industries, due to their high biocompatibility and biodegradability. In recent years, nanoliposome technology has become highly developed, offering real opportunities to food technologists in areas such as the controlled release and encapsulation of food ingredients and improvement of the stability and bioavailability of sensitive compounds. In this way, liposomes are being used as an advanced technology to carry active molecules to specific targets [11].

#### 2.2.2. Polymeric Nanoparticles

Most polymeric nanoparticles are known for their biodegradability and biocompatibility, constituting the most commonly used NPs in drug delivery systems [14–16]. This type of nanoparticle can be made from natural polymers, such as chitosan, or synthetic polymers, such as polylactides (PLA), poly (methyl methacrylate) (PMMA), or polyethylene glycol (PEG) [14]. They exhibit great potential for surface modification and have a good pharmacokinetic profile in that their size and solubility can be controlled during manufacture. Polymeric nanoparticles can be prepared by different methods, including two-step procedures based on emulsification, emulsification-solvent evaporation, emulsification-solvent diffusion, and emulsification–reverse salting-out. Additionally, there are methods such as one-step procedures involving nanoprecipitation methods, dialysis and supercritical fluid technology. Among the techniques used to analyse surface properties, we can find energy dispersive spectroscopy (EDS), zeta potential (*ζ*-potential), X-ray photoelectron spectroscopy (XPS), Fourier transform infrared spectroscopy (FTIR), and Raman. These techniques reveal the chemical composition of polymeric nanoparticle surface and surface functionalization. However, only by using microscopic techniques is it possible to identify morphology and shape. Finally, it is important to take into account that, in order to improve drug-loading efficiency and prolong drug release, the nature of polymer-drug interactions, as well as the polymer type and its physicochemical properties, must be considered [17].

#### 2.2.3. Metallic Nanoparticles

These include precious metals (gold or silver) and magnetic metals (iron oxide or cobalt and manganese doped ferrites). Metallic nanoparticles such as gold (Au) possess unique electronic and optical properties and are nontoxic and biocompatible, and their surface can be modified with other biomolecules due to their negative charge [18, 19]. A gold surface offers a fantastic opportunity to conjugate ligands such as proteins, oligonucleotides, and antibodies containing functional groups such as phosphines, thiols, mercaptans, and amines, which have a high affinity for the gold surface [20]. Gold nanoconjugates coupled with strongly enhanced localized surface plasmon resonance gold nanoparticles have applications in imaging techniques for the diagnosis of various diseases [21]. In fact, El-Sayed et al. established the use of gold nanoparticles (AuNPs) for cancer imaging by selectively transporting AuNPs into the cancer cell nucleus, thus highlighting the importance of these nanoparticles in biomedicine. In order to do this, they conjugated arginine–aspartic acid–glycine peptide and a nuclear localization signal peptide to a 30 nm AuNPs. The conjugated arginine–aspartic acid–glycine peptide targets *α*v*β*6 integrin receptors on the surface of the cell, whereas the lysine–lysine–lysine–arginine–lysine sequence associates with karyopherins (importins) in the cytoplasm, which enables translocation to the nucleus [22].

#### 2.2.4. Metal Oxide Nanoparticles

These NPs exhibit catalytic and antioxidant activities, chemical stability, optical properties, and biocompatibility, all of which make them suitable for several biomedical applications. The most widely used are iron oxide (Fe_3_O_4_), titania (TiO_2_), zirconia (ZrO_2_), and more recently, ceria (CeO_2_) [23]. For instance, titania nanoparticles are incorporated into medical implants due to the biocompatibility of their surface, and ceria nanoparticles are the object of increasing attention because of their catalytic and antioxidant capacity, which allows them to act as antioxidant and anti-inflammatory agents [2]. TiO2 is a widely studied material due to its biocompatibility, chemical stability, and optical properties, which endow it with important applications, for instance, as a biosensor [24]. Other metal oxide nanoparticles of increasing interest for their potential biomedical applications are cerium oxide (CeO_2_) nanoparticles or nanoceria. Nanoceria have the unique property of being able to switch between oxidation states [2], therefore enhancing their application in oxidative stress-related diseases. Cerium oxide nanoparticles have many defects on their surface, mainly O_2_ vacancies that result in a combination of coexisting cerium (IV) and cerium (III) oxidation states. This leads to a redox couple, which underlies nanoceria's catalytic activity. These characteristics endow nanoceria with great potential as a biological antioxidant. Other examples of metal oxide nanoparticles are porous silica (SiO_2_). The biomedical applications of these nanoparticles are increasing due to their unique properties, which include large specific surface area, pore volume, controllable particle size, and good biocompatibility. It is due to these properties that mesoporous silica nanoparticles have been investigated for their use in drug delivery in biomedicine and biosensors [25].

Studies have demonstrated the effectiveness of the use of other nanoparticles, such as zinc oxide (ZnO), in drug delivery and bioimaging. One important characteristic of ZnO nanoparticles is that their surface needs to be modified to protect them in biological systems, as they can be easily dissolved in water and acidic solutions. Furthermore, in order to use ZnO nanoparticles for fluorescence in imaging, they first need to be doped, as the ZnO bandwidth is in the UV region and UV light cannot penetrate tissues and can be harmful to cells and tissue [26].

#### 2.2.5. Ceramic Nanoparticles

These are inorganic compounds with porous characteristics that have recently emerged as vehicles for drugs. They are capable of transporting molecules such as proteins, enzymes, or drugs without swelling or compromising their porosity due to the external effects of pH or temperature [27]. The components most commonly used in ceramic nanoparticles are silica and aluminum. However, the core of these nanoparticles is not limited to these two materials; in fact, they can be composed of a combination of metallic and nonmetallic materials [28]. For instance, CeO_2_-capped mesoporous silica nanoparticles (MSN) have been developed to act as vehicles for drug delivery by releasing *β*-cyclodextrin into lung cancer cells [29].

There are a wide range of ceramic materials with multiple applications, including clay minerals, cement, and glass. Biocompatible ceramics, also known as bioceramics, are mainly used for the bone, teeth, and other medical applications. Bioceramics have good biocompatibility, hydrophilicity, osteoconductivity, biodegradability, and reabsorbability. The most widely used ceramic nanobiomaterials are calcium phosphate (CaP), calcium sulphate and carbonate, tricalcium phosphate (TCP), hydroxyapatite (HAP), TCP+HAP, bioactive glasses, bioactive glass ceramics, titania-based ceramics, alumina ceramics, zirconia ceramics, and ceramic polymer composites. All have been applied in nanomedicine, orthopedics, bone regeneration, dentistry, and tissue development, in addition to other biomedical uses in the human body [30].

#### 2.2.6. Quantum Dots

These are nanoparticles made of semiconductor materials with fluorescent properties. In general, quantum dots (QDs) consist of a semiconductor core (e.g., cadmium–selenium (CdSe), cadmium–tellurium (CdTe), indium–phosphate (InP), or indium–arsenate (InAs)), overcoated with a shell (e.g., zinc sulfide (ZnS)) to improve their optical and physical properties and to prevent leaking of the toxic-heavy metals [31]. These nanoparticles are the most used in bioimaging and biosensing strategies. However, this use requires them to be conjugated to biomolecules, such as proteins, peptides, or oligonucleotides, which enables them to bind to specific sites [32].

QDs' biocompatibility is essential for their biological and biomedical applications. In general, biocompatible QDs can be obtained through three different routes: (1) biomimetic synthesis, through either the use of artificial cellular structures or biomolecules (nucleic acids, peptides, proteins, and enzymes) as templates; (2) biosynthesis, using living organisms in bioreactors; or (3) modifying the surface of QDs derived from chemical synthesis. The biosynthetic approach provides a green pathway for preparing biocompatible QDs without generating toxic products or aggressive reaction conditions, while the surface modification approach can create a high QY on a large scale. One of the most important QDs are gold quantum dots (GQDs), which have similar properties to those of gold nanoparticles; however, unlike other QDs, they do not display fluorescence. Instead, they have colorimetric properties induced by surface plasmon resonance (SPR) depending on solvency, shape, particle size and ligand, surface functionalization, dielectric properties, medium, and agglomeration, which render them highly useful in biological system detection applications, such as DNA sequencing, hybridization assays, genetic disorders, flow cytometry, and immunoblotting [33]. In this sense, Lin et al. [34] synthesized GQDs and functionalized them with a peptide moiety containing a nuclear export signal and caspase-3 recognition sequences in order to use them as protease-mediated cytoplasm-nucleus shuttles for the dynamic monitoring of apoptosis. Once apoptosis is induced, the activated caspase-3 cleaves the functional peptide on GQDs, changing the subcellular distribution of GQDs, which are quantified as a function of time by the ratio of photoluminescence in the nucleus to that in the cytoplasm. GQDs function as molecular probes for real-time monitoring of cellular apoptosis, making them ideal for use in cancer.

#### 2.2.7. Carbon-Based Nanoparticles

These include fullerenes and nanotubes. Fullerenes are novel carbon allotropes with a polygonal structure made up exclusively of 60 carbon atoms [35]. Carbon nanotubes are normally manufactured from chemical vapor deposition of graphite. There are two classes of carbon nanotubes: single-walled (SWCNT) and multiwalled (MWCNT), the latter of which exhibits potent antimicrobial properties [36]. Carbon-based nanoparticles are considered of interest in biomedical applications due to their physical properties, including high electrical conductivity and excellent mechanical strength, but they are not biodegradable and require surface modifications, as they have a strong tendency to form large aggregates [37–39].

Carbon nanotubes (CNTs) have outstanding optical properties, which is why they are used as labeling and imaging agents [35]. In fact, CNTs have optical transitions in the near-infrared (NIR) region, which make them useful in biological tissue and cells, as NIR has lower excitation scattering and greater penetration depth [40]. Furthermore, fluorescence in the NIR region displays much lower autofluorescence than ultraviolet or visible ranges. For all these reasons, CNTs are potent imaging agents with higher resolution and great tissue depth for NIR fluorescence microscopy and optical coherence tomography. In this sense, Cherukuri et al. successfully monitored CNTs in phagocytic cells and in mice (administered intravenously) using NIR fluorescence [41]. However, several studies have reported cytotoxicity induced by CNTs. In this context, Yang et al. have analysed the behaviour of CNTs, such as agglomeration, cellular uptake, or their oxidant activity [42]. The controversial results regarding the biocompatibility of CNTs largely stem from the variability of CNTs (i.e., size, surface properties, charge, and functionalization) and testing subjects (i.e., *in vitro* vs. *in vivo*, types of cells, tissues, and animal models employed). In addition, increased cytotoxicity has often been attributed to incomplete removal of metal catalysts used to prepare CNTs [43]. Most *in vivo* studies using CNTs have shown them to be safe, as toxicity was not reported and there was renal clearance from the body, although small portions of CNTs were found in certain organs, such as the liver, spleen, and lungs [43]. In addition, several *in vitro* cell culture studies have indicated that the related cytotoxicity is more variable and more pronounced at the cellular level [44].

### 2.3. Toxicity Concerns

Nanoparticles offer many advantages to the field of medicine. However, the properties that make them so attractive can also contribute to their toxicological profile in biological systems (absorption, distribution, metabolism, and clearance). Toxicological concerns means that size, shape, surface chemistry, and chemical compounds need to be considered during the manufacture of nanoparticles [10, 45]. These concerns also have a bearing on cellular interactions, the endocytic pathway and the absorption process, and therefore, nanoparticles can exert mechanisms of cytotoxicity that interfere with cellular homeostasis.

The size of nanoparticles represents one of the crucial factors in their interaction with biological systems and is highly associated with their toxicological effects. Smaller NPs have a greater surface area per unit mass, which allows them to absorb large numbers of chemical molecules, making them more reactive in the cellular environment, thus increasing their toxicological effects [46]. *In vitro* studies have shown that nanoparticles below 10 nm are potentially harmful for the lungs due to the large surface area and possible nuclear penetration [47]. Less attention has been paid to the effect of NP shape, possibly due to the fact that most NPs are spherical. In general, the endocytosis of spherical NPs by cells is facilitated more than that of rod-shaped NPs and is relatively less toxic. For instance, gold nanorods show greater potential in cancer hyperthermia than conventional spherical gold nanoparticles, since they can cause breast cancer cell death due to their shape, among other reasons [48].

Some features related to the surface of NPs (surface chemistry and charge) are important in estimating their toxicity and are closely associated to uptake efficiency. For instance, the uptake of NPs made from hydrophobic polymers is greater than that of those made from hydrophilic polymers, which can enhance permeability and retention effect in tumor targeting [49]. Nanoparticle charge can also influence their uptake. Generally, positive NPs are internalized more readily by cells than negative NPs due to the negative charge of the cell membrane. Despite their bactericidal effects, silver nanoparticles have largely detrimental effects in biomedical applications. In this sense, it has been demonstrated that the surface chemistry and charge of these nanoparticles make them susceptible to a greater internalization, therefore rendering them more toxic [50, 51].

The toxicity of NPs also depends on the chemical components on their surface. Some metal oxides, such as zinc oxide (ZnO), manganese oxide (Mn_3_O_4_), or iron oxide (Fe_3_O_4_), have intrinsic toxicity potential [10, 52]. NPs made from these metal oxides can induce cytotoxic effects that can be reversed by modifying their surface. However, these adverse effects are often very useful in cancer cell therapies [10]. Another chemical component investigated in the context of nanoparticle toxicity is silver (Ag), as it can be widely used and is easily found in the environment. The cytotoxic effects of silver nanoparticles (AgNPs) include induction of ROS and oxidative stress, DNA damage, and apoptosis [53].

The most common route of administration of NPs is intravenous injection. Once they reach the vascular system, they are distributed to various organs and tissues. The physicochemical properties of NPs determine their distribution patterns. Nevertheless, it seems clear that size and surface are the key factors for improved and long-lasting biodistribution. In this sense, smaller nanoparticles (1–20 nm) tend to infiltrate the organs more than larger ones, and surface modification with hydrophilic polymers such as polyethylene glycol (PEG) enhances their blood circulation time. Regarding biological clearance, renal excretion represents the main route of elimination of exogenous material, such as NPs [54]. Nevertheless, while NPs are in the bloodstream, they can be cleared by other routes, such as disintegration through protein absorption or opsonization-mediated removal by the mononuclear phagocytic system (MPS) [55]. Metallic NPs tend to accumulate in the spleen, liver, and lymph and are able to remain for months due to their nonspecific uptake by MPS [56].

### 2.4. Advantages of Nanoparticles and Their Potential Role in Oxidative Stress Treatment

Nanoparticles have many potential benefits for medicine, and current clinical applications include diagnosis and therapy. In particular, NPs offer several advantages that make them suitable as drug delivery systems [1–3]. These advantages include (i) easy uptake by the cells due to their small size; (ii) a large surface area-to-volume ratio, which controls the absorption and sustained release of drugs; and (iii) targeted delivery to specific sites. Taking into account these features and considering them from a therapeutic perspective, perhaps the greatest potential of NPs is their use against diseases involving oxidative stress, such as atherosclerosis [6]. Currently, one of the strategies against ROS—whose aim is to maintain and optimize the antioxidant system—is the use of exogenous antioxidants such as vitamin C, vitamin E, or other antioxidants such as *β*-carotene. Nevertheless, these molecules have a limited effect, since they are not directly internalized by the cells and sometimes do not reproduce the same results in clinical trials [57, 58]. Consequently, the development of other exogenous substances with better antioxidant properties is necessary. In this sense, NPs made from metal oxide, such as cerium oxide (CeO_2_) or yttrium oxide (Y_2_O_3_), and those that are carbon-based, such as fullerenes, have generated much interest because of their radical-quenching and catalytic properties [59]. For instance, Ciofani et al. found out that cerium nanoparticles or nanoceria were capable of scavenging intracellular ROS, reporting a reduction of 25% to 50% of basal ROS in cells exposed to oxidative stress caused by H_2_O_2_ [60]. Therefore, it appears that these types of NPs can be used as ROS scavengers, thus providing a novel strategy against oxidative stress.

It is important to take into account the limitations of the use of small-molecule natural and synthetic antioxidants which include low solubility, poor bioavailability, and their lack of specificity. In this sense, polymeric nanoparticles can be used to encapsulate or incorporate small molecules to provide protection from degradation or to aid in the absorption and distribution of natural antioxidants. Nanoparticles can provide higher solubility to compounds with poor water solubility and enhanced surface functionalization to yield target specificity. Some NPs are known to have prooxidant properties [61], but this action can be avoided by using biodegradable carrier molecules, such as albumin or poly (lactic-co-glycolic) acid (PLGA), which can be broken down by lysosomal or hydrolytic degradation of the matrix polymers [62].

NPs can be carriers of antioxidants, for instance, SOD-containing nanoparticles. In this sense, the application of nanomedicine in ROS-mediated pathologies has dramatically advanced strategies to promote the scavenging of free radicals under oxidative stress, including target specificity, increased cell membrane permeability, and the use of catalytic scavengers. The obvious advantage of using a catalytic ROS scavenger is that the compound is not depleted during the reaction and can potentially scavenge numerous ROS molecules, which can enhance their potency with a lower dose. Endogenously, cells use SOD to catalyze the neutralization of O_2_^•−^ to O_2_ and H_2_O_2_. Nanoparticles can be engineered with recombinant SOD conjugation to allow effective cellular delivery of the enzyme under oxidative stress conditions, while protecting the enzyme and avoiding its degradation in the serum [63]. The conjugation of SOD to nanoparticles also promotes blood–brain barrier permeability, which allows these nanoparticles to be used in the context of ischemia/reperfusion injury in the brain. Upon reaching the cells, the nanoparticle is endocytosed, and the enzyme catalyzes the degradation of O_2_^−^. Reddy and Labhasetwar used SOD-conjugated poly (D,L-lactic-co-glycolic acid) (PLGA) nanoparticles to treat ischemia/reperfusion injury in the brains of rats, achieving sustained SOD delivery that enhanced the survival rate and improved their neurological function. Furthermore, an infusion of SOD nanoparticles during reperfusion reduced the infarct size by 65% compared with a saline control and 40% compared with SOD delivered in solution. Chen et al. recently engineered silica nanoparticles conjugated with recombinant Cu/Zn SOD containing a His-tag domain for attachment to the nanoparticle and a human immunodeficiency virus (HIV) transactivator protein (TAT) domain which allows enhanced transmembrane delivery [64].

Other interesting studies have assessed the use of platinum nanoparticles. Platinum has been used clinically as a chemotherapeutic agent—for example, cisplatin—and in chemistry as a catalyst for hydrogenation and oxidation reactions. It has been shown to catalytically convert O_2_^−^ to H_2_O_2_ and H_2_O_2_ to H_2_O and O_2_, which makes it an attractive candidate as a SOD/catalase mimetic for the treatment of oxidative stress-related diseases [65].

Another possibility for antioxidant effects is the use of cerium nanoparticles, which possess catalytic properties similar to those of platinum nanoparticles due to their ability to convert O_2_^−^ to O_2_, to generate Ce^3+^ from Ce^4+^, and to then autoregenerate Ce^4+^ from the reduction of Ce^3+^ or by reacting with HO^•^. Ceria have also been found to catalyze the degradation of H_2_O_2_ [66], showing the multifaceted mechanism of their antioxidant properties. Furthermore, nanoceria have also been shown to have anti-inflammatory effects by decreasing NO production from macrophages in mouse cells through a downregulation of iNOS [67]. In another study, ceria were shown to scavenge for ONOO^−^, a potent RNS generated by the reaction of O_2_^−^ with NO [68]. These properties make ceria particularly useful in chronic ROS-mediated inflammatory diseases, where scavenging of NO generated from iNOS in macrophages can halt further inflammatory damage. However, *in vitro* studies are contradictory, as they provide conflicting evidence of ceria toxicity in different cell lines, perhaps attributable in part to the size and surface area of the ceria particles, with larger particles exhibiting greater toxicity. For example, Estevez et al., [69] evaluated the effects of ceria in an ischemic model of mouse hippocampal brain slices and found approximately a 50% reduction in cell death, probably due to a marked decrease in the levels of ROS and ONOO−. Ceria can be readily taken up by the cells; however, their tendency to form aggregates in the cytoplasm limits their antioxidant properties [70].

There is another possible application of H_2_O_2_-sensitive nanoparticles in biomedicine. Importantly, ROS production is ubiquitous under physiological conditions, and therefore, a compound that scavenges ROS in a tissue-specific way (e.g., in oxidative tissue) would increase the antioxidant efficacy enormously. In this sense, the development of molecules in response to oxidative stress makes them a useful target for the delivery of antioxidants. For example, Lee et al. [71] developed an antioxidant nanoparticle that is insensitive to H_2_O_2_. This compound consists of a copolyoxalate-containing vanillyl alcohol (PVAX) particle, and its structure contains peroxalate ester linkages that degrade upon reaction with H_2_O_2_. These effects induce the release of the antioxidant vanillyl alcohol, which decreases ROS production and the inflammatory process associated with it. Furthermore, vanillyl alcohol can also downregulate the expression of cyclooxygenase-2 (COX-2) and iNOS [72].

Another approach for the use of nanoparticles in biomedicine is the use of pH-sensitive nanoparticles. In this sense, it has been described that nitroxyls such as TEMPO are stable radical compounds that are able to scavenge ROS and to form two nonradical species. Another important characteristic of these compounds is their ability to partially mimic SOD, due to their capacity to self-regenerate under oxidative stress conditions. This potential effect of nitroxyls *in vitro* cannot be replicated in *in vivo* treatments because they are hipotensive agents and therefore affect the cardiovascular system, especially in a model of ischemia/reperfusion (I/R). One interesting study by Marushima et al. has identified a micelle nanoparticle with encapsulated 4-amino-TEMPO which is able to protect nitroxyl compounds in vivo [73]. This nanoparticle was shown to decrease infarct size in an animal model of acute cerebrovascular I/R injury. Furthermore, it did not change blood pressure, unlike the free TEMPOL, which decreases blood pressure. In addition, this compound has an *in vivo* half life longer than that of TEMPOL.

Some NPs are able to release their content in response to a decrease in pH levels. This is the case of radical-containing-nanoparticle (RNP) micelles, which are sensitive to pH. They are able to build polymers under mildly acidic conditions, thus allowing leakage of the TEMPO molecules to the ischemic zone. This therapeutic possibility was developed for the treatment of chronic neurodegenerative disease [74]. The micelle is broken open by the stomach's low pH, and polymer molecules with covalently linked TEMPOL are then absorbed. Overall, the protection and target specificity offered by the micelle's encapsulation of RNPs show great promise for their application as antioxidant therapies for oxidative stress-mediated disease.

Diamond nanoparticles (DNPs) are another interesting type of nanoparticle. DNPs are obtained by explosive detonation and can be treated under Fenton conditions (FeSO_4_ and H_2_O_2_ at acidic pH) to obtain purer DNP samples with a small average particle size (4 nm) and a large population of surface OH groups (HO-DNPs). Fenton-treated HO-DNPs can support gold and platinum nanoparticles of less than 2 nm. In this sense, Martin et al. [75] demonstrated that the resulting materials (Au/HO-DNP and Pt/HO-DNP) exhibit a high antioxidant activity against ROS induced in a hepatoma cell line. Furthermore, both Au/HO- and Pt/HO-DNPs exhibited good biocompatibility, exhibiting a two-fold higher antioxidant activity with respect to that of glutathione. In another study with ceria-supported gold nanoparticles, they exhibited peroxidase activity and acted as radical traps. In fact, Au/CeO(2) showed a remarkable biocompatibility with two human cell lines (Hep3B and HeLa), demonstrated by measuring cellular viability, and proliferation and lack of apoptosis. Au/CeO(2) exhibited higher antioxidant activity than glutathione, the main cytosolic antioxidant compound, and its CeO_2_ carrier. Overall, these results highlight the potential of implementing well-established nanoparticulated gold catalysts with remarkable biocompatibility in cellular biology. A study by Li et al. [76] demonstrated the superoxide-scavenging ability of ceria nanoparticles, reporting that nanoceria greater than 5 nm, with different shapes, and with a negligible Ce^3+^/Ce^4+^ ratio can acquire remarkable superoxide-scavenging abilities through electron transfer.

Finally, we would like to highlight the importance of the development of mitochondria-directed nanoparticles. In general, there are many diseases which are related to mitochondrial dysfunction and, therefore, to high ROS production and oxidative stress. Consequently, targeting mitochondria to deliver antioxidants can decrease ROS formation and maintain cell function [77–79]. For example, triphenylphosphonium (TPP) is a molecule capable of crossing cell membranes and can accumulate in mitochondria due to its lipophilic cation. In one study, Marrache and Dhar designed a combination of conjugated PLGA-b-poly (ethylene glycol) (PEG) nanoparticles with TPP-enhancing antioxidant protection and which improved the site-directed delivery of mitochondria-targeting chemotherapeutics [80]. In said study, PLGA-b-PEG-TPP nanoparticles were loaded with curcumin, a known antioxidant with important therapeutic effects in different diseases, in order to deliver them more effectively to human neuroblastoma cells. This alternative therapy for mitochondrial-directed nanoparticles is a novel approach for the site-specific delivery of ROS scavengers to combat oxidative stress and mitochondrial dysfunction in multiple diseases and therefore reduce the risk-to-benefit ratio.

In conclusion, as a whole, the aforementioned evidence endorses nanoparticles as a promising antioxidant therapy, either for use as carriers of antioxidants or due to their own antioxidant activity.

## 3. Targeting Vascular Oxidative Stress Using Nanoparticles

Oxidative stress is characterized by an increase of reactive oxygen and nitrogen species (RONS) derived from the physiological process of cellular oxidation. In healthy conditions, the antioxidant system counterbalances an excess of RONS in order to maintain the equilibrium of the organism. The imbalance in favour of oxidative stress is related to several pathological conditions, such as vascular dysfunction, characterized by impaired endothelial NO bioavailability and an impairment in vasodilation response, and proinflammatory states. However, the interaction among vascular dysfunction, inflammation, and oxidative stress is not fully understood.

Sources of reactive oxygen species (ROS) in the vascular wall include NADPH oxidase (Nox) [81], uncoupled endothelial NO synthase (eNOS) [82], xanthine oxidase (XO) [83], and mitochondrial respiratory chain enzymes [84]. Under physiological conditions, Nox prevails, and some interactions have been described between Nox and other oxidant mechanisms. In line with this, NADPH oxidase is related to an increase in the activity of xanthine oxidase, eNOS uncoupling, and mitochondrial ROS production [84]. It is noteworthy that angiotensin II (AT II) is related to ROS production at the vascular level by increasing the expression of Nox [85] and xanthine oxidase [86] and reducing the antioxidant system thioredoxin [87]. Therefore, in the present review, we also focus on the renin-angiotensin system, since it is implicated in the vascular complications related to oxidative stress.

Blood flow exerts a frictional force on vascular endothelial cells, namely, hemodynamic shear stress, which is related to the release of ROS [88]. Under physiological conditions (regular flow pattern), shear stress releases endothelial nitric oxide (NO) from L-arginine by eNOS. NO is a potent vasodilator [89] that inhibits platelet adhesion and aggregation [90], vascular smooth muscle proliferation [91], and exerts antiatherogenic effects [92]. Superoxide anion (O_2_^−^), the most common ROS, interacts with NO to generate peroxynitrite (ONOO^−^), considered a reactive nitrogen species (RNS) [93]. Although this does not happen under normal conditions, because the production of ROS is limited, in conditions of vascular oxidative stress, the increase of ROS reduces the bioavailability of NO. The vascular wall also contains antioxidant systems, such as superoxide dismutase (SOD), catalase, glutathione peroxidases, thioredoxin system, and peroxideroxins.

In this sense, we will now describe each oxidant or antioxidant system, and we summarize some of the research about nanoparticles which affect these systems at a vascular level.

### 3.1. NADPH Oxidase in the Vascular Wall

**Figure 2:**
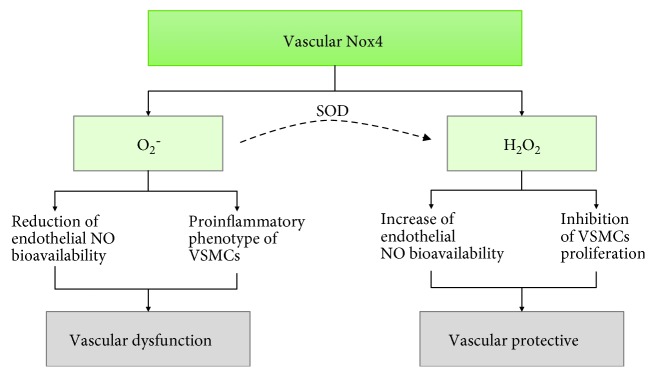
The Nox 4 paradox. Vascular Nox 4 generates superoxide anion (O_2_^−^) and hydrogen peroxide (H_2_O_2_). Depending on which via predominates, Nox 4 activation can be damaging or protective. VSCMs: vascular smooth muscle cells.

NADPH oxidases use NADPH to reduce O_2_ to O_2_^−^. Nox 1, 2, 4, and 5 are expressed in the vascular wall; Nox2 [94] is located mainly in endothelial cells and Nox1 [95] in vascular smooth muscle cells (VSMC), whereas Nox 4 and Nox 5 can be found in both cell types [96]. Nox family enzymes are implicated in some vascular diseases, such as hypertension, atherosclerosis, and vascular diabetic complications, and as a result, their role in vascular pathologies has received much attention. Nox 2 gene expression is inducible and increases in response to AT II in some vessels, such as the aorta or resistance arteries [96]. Nox 1 expression is induced by prostaglandin F2alpha, platelet-derived growth factor, and also by AT II in vascular smooth muscle [96]. However, the effects of AT II on the activation of Nox 4 expression are contradictory [96]. Little is known about the activation of Nox 5 at the vascular level. On the contrary, Nox 4 is the most abundant isoform in the vascular system, and NADPH oxidase activity depends mainly on its expression under resting conditions [96, 97]. Nox 4 releases hydrogen peroxide (H_2_O_2_) in preference to O_2_^−^ [98], which can offer a protective role against atherogenesis, as H_2_O_2_ does not interact with NO to form ONOO^−^ [99–104]. Moreover, H_2_O_2_ activates eNOS [98, 105] and inhibits vascular smooth muscle cell proliferation, thereby preventing vascular inflammation and remodelling [100, 106]. However, other studies have demonstrated a detrimental role of Nox 4. For instance, Lozhkin et al. observed an increase in the expression and activity of Nox 4 during aging, which enhanced cellular and mitochondrial oxidative stress and vascular dysfunction, leading to a proinflammatory phenotype in VSMC [107]. Similarly, in experimental models of ischemic stroke [108, 109], cardiac hypertrophy [110], or diabetic cardiomyopathy [111], the role of Nox 4 is not beneficial.  Figure 2 shows the paradoxical effects of vascular Nox 4.

One study using iron oxide nanoparticles (Fe_2_O_3_-NPs) demonstrated that, due to overexpression of Nox 4, Fe_2_O_3_-NPs disturbed the balance between oxidants and antioxidants, resulting in oxidative myocardial damage [112]. Other nanoparticles have shown effects on NADPH oxidases; for example, Sun et al. [113] demonstrated that AgNPs decreased cell viability, induced ROS generation, and led to early apoptosis in human umbilical vein endothelial cells through upregulation of Nox 4 protein expression. Abe et al. [114] and Kim et al. [115] described the properties of platinum-loaded tungsten oxide (WO_3_-Pt) nanoparticles and their interaction with organophosphorous compounds [116]. NADPH contains phosphorous, which is an excellent substrate for WO_3_-Pt nanoparticles, and has recently been proposed as a NADPH oxidase biomimetic with potential as an antitumor agent [117].

The role of Nox 4 in vascular function continues to be a subject of controversy; some studies report a protective role against atherogenesis, while others show the contrary. Further research is needed to understand better the effect of nanoparticles targeted to Nox in the vascular system.

### 3.2. Uncoupled eNOS

**Figure 3:**
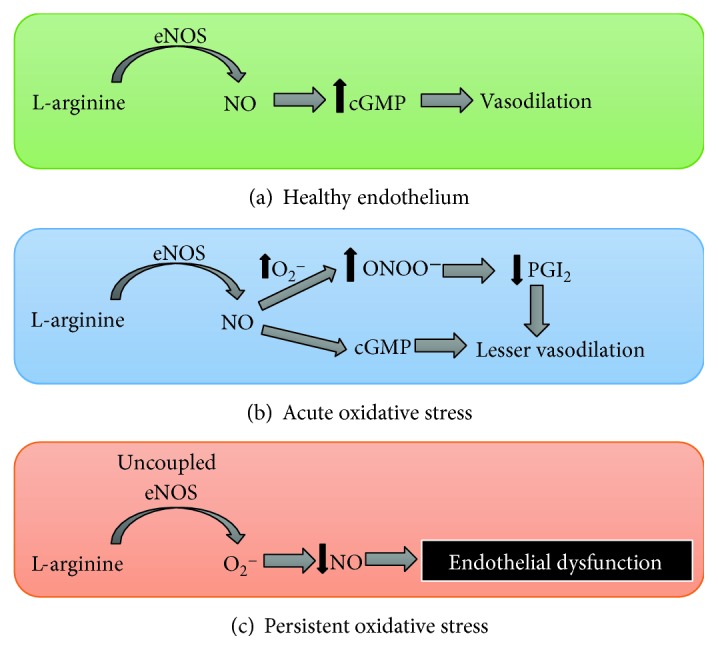
Endothelial dysfunction due to oxidative stress. The image shows how vascular oxidative stress leads to endothelial dysfunction. (a) represents the production of nitric oxide (NO) from L-arginine by endothelial nitric oxide synthase (eNOS) in a healthy endothelium, where levels of superoxide anion (O_2_^−^) are low. NO diffuses from the endothelium to the vascular smooth muscle, where it activates soluble guanylate cyclase (sGC), which increases the levels of guanosine 3′,5′ cyclic monophosphate (cGMP), thus leading to vasodilation. (b) represents acute vascular oxidative stress, with an increase in O_2_^−^ production, followed by an increase in peroxynitrite (ONOO^−^) levels. ONOO^−^ produces endothelial dysfunction by directly reducing the NO available for activating sGC and by reducing prostaglandin I_2_ (PGI_2_) content via nitration PGI_2_ synthase. If oxidative stress is persistent (c), eNOS becomes uncoupled, producing O_2_^−^ instead of NO and aggravating endothelial dysfunction.

NO is synthesized from L-arginine by the action of NOS [118]. There are three isoforms of NOS: endothelial (eNOS), neural (nNOS), and inducible (iNOS) [119]. The third of these is activated under inflammation, and the amount of NO it produces is greater than that generated by the other two isoforms [120]. eNOS converts L-arginine into L-citrulline and NO in the presence of cofactors such as nicotinamide adenine dinucleotide phosphate (NADPH), Ca^2+^/calmodulin (CaM), flavin adenine dinucleotide (FAD), flavin mononucleotide (FMN), and tetrahydrobiopterin (BH4) [121]. NO activates soluble guanylate cyclase (sGC), which stimulates the conversion of guanosine-5-triphosphate (GTP) to guanosine 3′,5′ cyclic monophosphate (cGMP), leading to a reduction of cytosolic calcium and vasodilation [122]. Decreased bioavailability of NO is the main cause of endothelial dysfunction. Vascular oxidative stress can contribute to the uncoupling of eNOS [123, 124] and, therefore, endothelial dysfunction. Uncoupled eNOS exhibits NADPH oxidase activity and produces O_2_^−^. The main causes of this uncoupling are deficiency of L-arginine, BH_4_, or eNOS S-glutathionation [123, 125]. Deficiency of L-arginine can be related to an increase in arginase activity in blood vessels [126–128], and deficiency of BH_4_ is due to an increase in its oxidation [129]. eNOS can also be uncoupled by S-glutathionylation, observed in patients with hypertension, and increased levels of AT II [130]. In this respect, oxidative stress contributes to endothelial dysfunction: firstly, because NO is inactivated by O_2_^−^ to form ONOO^−^, and secondly, because persistent oxidative stress causes eNOS to uncouple (Figure 3).

Regarding the effects of nanoparticles on NOS, Ramirez-Lee et al. [131] evaluated the effect of AgNPs using isolated perfused hearts from hypertensive rats. They reported that NO derived from both eNOS and iNOS was reduced, leading to increased vasoconstriction and myocardial contractility. Although the reduction of NO from iNOS could be considered beneficial due to its anti-inflammatory effect, the authors concluded that AgNPs intensify the hypertension. As we have discussed above, NO maintains vascular homeostasis, increases vascular endothelial growth factor, and prevents platelet adherence and leukocyte chemotaxis. Due to these effects, it is used to promote physiological angiogenesis in the treatment of peripheral arterial diseases. However, the ischemic event during peripheral ischemia produces O_2_^−^ and diminishes the bioavailability of NO by forming ONOO^−^. An interesting recent study [132] developed a hybrid molecule consisting of a copolymer poly (lactic-co-glycolic acid) (PLGA) nanoparticle loaded with SA-2 and which contains both antioxidant and NO donor functionalities and provides a sufficiently therapeutic level of NO to treat peripheral arterial diseases.

### 3.3. Xanthine Oxidase in the Vascular Wall and Angiotensin II

**Figure 4:**
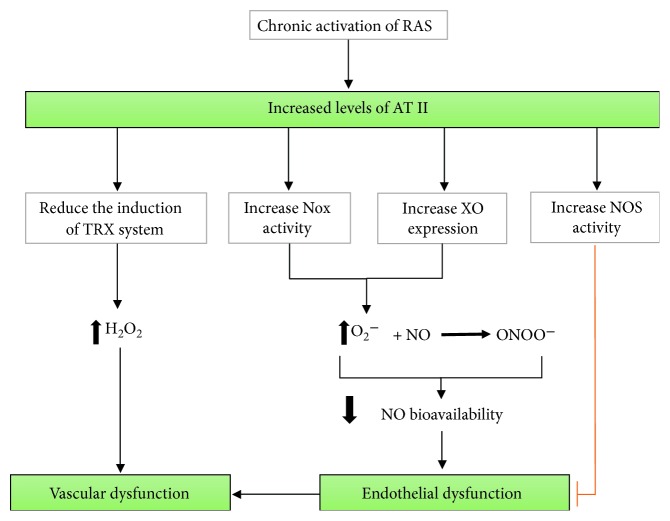
Chronic activation of the renin-angiotensin system (RAS) contributes to oxidative stress and vascular dysfunction. Increased levels of angiotensin II (AT II) lead to endothelial dysfunction through AT II receptor 1 (AT1R) activation, which in turn induces vascular oxidative stress by increasing NADPH oxidase (Nox) activity and xanthine oxidase (XO) expression. Both enzymes produce superoxide anion (O_2_^−^), which scavenges nitric oxide (NO) by forming peroxynitrite (ONOO^−^), consequently decreasing NO bioavailability and causing endothelial dysfunction. Moreover, AT II can undermine the induction of the antioxidant system thioredoxin (TRX), enhancing levels of H_2_O_2_ and contributing to vascular oxidative stress. H_2_O_2_ is the most stable and abundant ROS which, as a signalling messenger, maintains physiologic vascular homeostasis, but its overproduction is related to vascular dysfunction. In contrast, AT II receptor 2 (AT2R) activation can counteract the lesser NO bioavailability induced by vascular oxidative stress via eNOS phosphorylation, thereby increasing its activity.

Xanthine oxidase forms O_2_^−^ and H_2_O_2_ [133–136]. Experimental studies have demonstrated that its expression is increased in response to AT II [83], and chronic activation of the renin-angiotensin system (RAS) may contribute to vascular xanthine oxidase activation [86]. The activation of AT II receptor 1 (AT1R), the high affinity receptor for AT II [137], also induces upregulation of NADPH oxidase activity, increasing O_2_^−^ [85, 138], which scavenges NO to form ONOO^−^ and consequently diminishes NO bioavailability, leading to endothelial dysfunction. On the contrary, AT II receptor 2 (AT2R), located in the endothelium, enhances phosphorylation of eNOS, thereby increasing its activity [139]. In this way, AT2R regulates O_2_^−^ production in the opposite manner to AT1R [139, 140] and balances the prooxidative function of AT1R (Figure 4). Therefore, patients with chronic hypertension and activated RAS could benefit from nanoparticles that are capable of downregulating AT1R or upregulating AT2R, though as far as we know, no studies have investigated this so far. Nevertheless, some researchers have designed nanoparticles carrying AT II; for instance, Hennig et al. demonstrated that AT II-coupled nanoparticles can be used to establish high affinities to cells with an overexpression of AT1R [141].

The best characterized antihypertensive peptides—achieved by inhibiting angiotensin-converting enzyme (ACE)—are Ile-Pro-Pro (IPP) and Val-Pro-Pro (VPP). However, they are compromised by their low oral bioavailability, which is mainly due to their gastrointestinal degradation. Consequently, the use of nanoparticles as carrier systems encapsulating these peptides could prevent their proteolysis and enhance their systemic uptake. Yu et al. [142] tested poly-(lactic-co-glycolic) acid (PLGA) nanoparticles (PLGANPs) as an oral delivery system for antihypertensive small peptides in a model of spontaneously hypertensive rats. The authors concluded that PLGANP was a potential therapeutic treatment for hypertension.

### 3.4. Effect of NO on Mitochondrial Respiratory Chain Enzymes

Mitochondria are the main source of O_2_^−^ [143]. NO binds to cytochrome oxidase (COX) and inhibits electron transfer to O_2_ [144]. The enzymes of the electron transfer chain show varying sensitivity to NO [145]. If exposure to NO is prolonged, the activity of NADH dehydrogenase is inhibited at mitochondrial complex I [146]. NO reacts with ubiquinol, oxidizing it to the respective semiquinone, which forms O_2_^−^ by autoxidation to ubiquinone. O_2_^−^ reacts with NO and forms ONOO^−^. O_2_^−^ can also dismute to H_2_O_2_ catalyzed by MnSOD [147]. The main mitochondrial antioxidant defence is MnSOD, and dismutation of O_2_^−^ by this enzyme occurs at a lower magnitude to the formation of ONOO^−^. Consequently, ONOO^−^ is elevated at high levels of NO. mtNOS is another NOS isoform present in the mitochondria [148, 149], but classic NOS isoforms are mainly responsible for cytosolic NO concentration. NO can spread to mitochondria, but at a very low concentration, for two reasons: first, because its concentration is low at physiological conditions; and second, because NO binds to cytosolic compounds. However, when iNOS is activated, as occurs during inflammation, levels of NO increase, as does the formation of ONOO^−^ [150]. Therefore, NO plays an important role in the mitochondrial oxidative stress occurring in some pathological conditions, such as atherosclerosis [151]. Some nanoparticles, such as nanoceria, have demonstrated their ability to reduce the expression of iNOS, thus exerting anti-inflammatory effects [67, 152]. Treatment of cultured cardiomyocytes with nanoceria has been shown to result in significant inhibition of cigarette smoke extract-induced ROS production [152].

### 3.5. Endothelial Dysfunction due to Oxidative Stress

The endothelium plays an essential role in vascular homeostasis and releases relaxing factors, such as NO, prostacyclin (PGI_2_), or endothelium-derived hyperpolarizing factor (EDHF) [153]. Endothelial dysfunction is the first phase in the development of cardiovascular diseases. As already explained, the main cause of endothelial dysfunction is a decrease in NO bioavailability [154]. It is well known that oxidative stress contributes to endothelial dysfunction, as we have already described (see Figures 2 and 3). Excess of ROS generates ONOO^−^, which can reduce vasodilation by two mechanisms: first, by directly reducing the NO available for activating the cGC in the vascular smooth muscle, and second, by reducing prostaglandin I_2_ content via nitration PGI_2_ synthase [155]. Consequently, endothelial dysfunction represents an imbalance between vasodilator and vasoconstrictor agents released by the endothelium, reducing the capacity of vasodilation or increasing the response to vasoconstrictor agonists.

Recent evidence suggests that some kinds of nanoparticles, such as silica nanoparticles, affect vasodilator function depending on their charge and size [156]. Silica nanoparticles were shown to attenuate vasodilation in the aorta, which was partially restored using SOD [157], implicating oxidative stress as the mechanism responsible. In the same line, Guo et al. reported that silica nanoparticles induced endothelial dysfunction through mitochondrial dynamics and alteration of biogenesis [158]. However, on the other hand, ceria nanoparticles seem to restore endothelial-dependent vasodilation. Minarchick et al. investigated the effects of nanoceria on vascular reactivity in a rat model of hypertension and concluded that they decreased the microvascular dysfunction and oxidative stress associated with hypertension [159]. Indeed, nanoceria can be considered a promising type of nanoparticle. They contain a high density of O_2_ vacancy in their structure, which confers them the ability to store O_2_ during the lean phase and to return O_2_ to metal particles during the oxygen-rich phase. This mechanism is known as the O_2_ storage capacity of ceria [2]. CeO_2_NPs could have cardiovascular-protector effects that render them controllers of endothelial inflammation. In line with this, studies have demonstrated nanoceria are SOD mimetic *in vitro* [160] and antioxidant and anti-inflammatory effects in the murine myocardium [161]. In contrast, other studies have reported a decrease in vascular function after nanoceria incubation due to an increase in ROS generation [162, 163]. It is likely that these contradictory effects are due to different basal levels of ROS in the animal models used.

Some studies have concluded that the effects of nanoparticles on endothelial function depend on the concentration and composition of the particles. CeO_2_NPs or nanoceria provoke a very slight inflammatory response in human aortic endothelial cells and seem to be rather benign in comparison with Y_2_O_3_ and ZnO nanoparticles [164, 165]. In light of all the above, further investigations are needed to assess the role of different nanoparticles in vascular reactivity, endothelial dysfunction, and toxicity.

### 3.6. Vascular Antioxidant Systems

As we have previously indicated, SOD produces H_2_O_2_ as a result of the dismutation of O_2_^−^. There are three isoforms of SOD: Cu/Zn SOD or SOD1, located at cytoplasm and mitochondria intermembrane space; SOD2, expressed in the mitochondrial matrix; and SOD3, which is extracellular and largely expressed in the vascular wall [166]. Although the role of SOD is antioxidant, it is worth noting that the capacity of the downstream enzymes to degrade H_2_O_2_ influences the oxidative balance. In line with this, studies analysing the effect of SOD on atherogenesis have concluded that moderate levels of SOD reduce ROS and that elevated levels induce oxidative damage and increase levels of proaterogenic molecules [167–169].

The enzymes that decompose H_2_O_2_, the most stable and abundant ROS [170], are catalase, glutathione, and thioredoxin peroxidases, and the correct functioning of these systems is vital, since an excess of H_2_O_2_ can cause vascular injury. There is evidence of a dual role of ROS: on one hand, they are signalling messengers and maintain physiologic vascular homeostasis; on the other, ROS excess is related to vascular dysfunction, such as that which occurs in hypertension, atherosclerosis, diabetes, or acute coronary syndrome [171, 172]. Catalase is an important antioxidant enzyme located in peroxisomes [173], transforming H_2_O_2_ into O_2_ and water and playing a central role against oxidative stress. Cerium nanoparticles exhibit CAT-mimetic activity, and this effect depends on their surface area, with the smallest ones proving to be the most active [174].

Glutathione peroxidases (GPx) are an enzyme family with peroxidase activity. GPx reduce H_2_O_2_ and lipid hydroperoxides to water and their corresponding alcohols, where reduced glutathione (GSH) is the main electron donor. GPx oxidizes GSH to form glutathione disulphide (GSSG), a reaction that is reversed by glutathione reductase, a NADPH-dependent enzyme [175]. This enzyme family protects against oxidative damage and represents the major antioxidant system within many cells [176]. The antioxidant property of glutathione lies in the presence of thiols, molecules that contain a sulfhydryl (SH) side chain group. Thiols reduce RONS by accepting their unpaired electron, which is essential for maintaining cellular reduction–oxidation (redox) status in favour of the former. There are several isoforms; GPx1, the most abundant in mitochondria and cytoplasm, is expressed in red blood cells, and its low activity has been related to cardiovascular risk [177].

The thioredoxin (TRX) system is integrated by NADPH, thioredoxin reductase, and thioredoxin, and its function is to regulate the equilibrium between protein dithiol and disulphide through disulphide reductase activity. The TRX system provides electrons to peroxiredoxins (thiol-dependent peroxidases) in order to remove RONS [178], and the reduced TRX peroxidase can scavenge H_2_O_2_ [179]. Regarding the TRX system's role in vascular function, both endothelial and smooth muscle cells express TRX, but the regulatory mechanism involved in its expression seems to differ. In the endothelium, it appears that TRX is a ROS-inducible protein, since treatment with H_2_O_2_ increases its expression [180, 181], whereas, in vascular smooth muscle cells, TRX is related to cell proliferation, and its induction is not regulated by ROS [180]. Treatment with AT II reduces the induction of TRX, contributing to oxidative stress in hypertension [87]. Moreover, TRX increases in response to an excess of NO due to activation of iNOS, representing a mechanism against vascular inflammation, nitrosative stress, and atherosclerosis [182].

Peroxiredoxins are a family of proteins that regulate levels of H_2_O_2_ by using TRX as an electron donor, and their function depends on the reduced forms of TRX and glutathione [183]. The peroxiredoxin 4 is capable of scavenging intracellular ROS from the endoplasmic reticulum, and oxidative stress and endoplasmic reticulum stress have been demonstrated to contribute to the onset and development of the inflammation that accompanies vascular disease, such as atherosclerosis [184, 185]. Another family with atheroprotective properties and anti-inflammatory effects—exerted by degrading H_2_O_2_—is the paraoxonase (PON) family, which is composed of three members: PON1, PON2, and PON3. PON2 and PON3 are expressed in the vascular wall. PONs also neutralize homocysteine thiolactatone, which has been related to vascular damage and atherogenesis [186].

**Figure 5:**
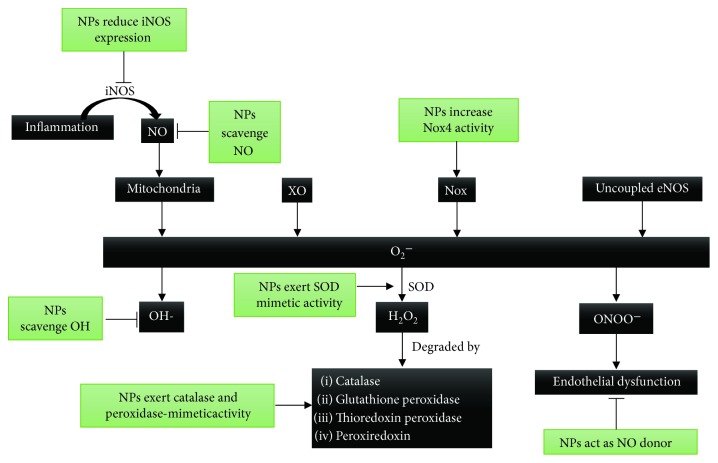
Effects of nanoparticles on the main mechanisms of vascular oxidative stress and antioxidant systems. Mitochondrial respiratory chain enzymes, xanthine oxidase (XO), NADPH oxidase (Nox), and uncoupled endothelial NO synthase (eNOS) are the main sources of superoxide anion (O_2_^−^) in the vascular wall. O_2_^−^ can produce hydroxil radical (OH), hydrogen peroxide (H_2_O_2_), and peroxynitrite (ONOO^−^). The enzymes that decompose H_2_O_2_ are catalase, glutathione, thioredoxin peroxidase, and peroxiredoxin. In inflammation, the induction of iNOS produces high levels of NO which react with mitochondrial respiratory chain enzymes and increase O_2_^−^ production. Some nanoparticles (NPs), such as nanoceria, have demonstrated the ability to reduce the expression of iNOS. Moreover, nanoceria can scavenge both NO and OH, thus proving to be anti-inflammatory and antioxidant agents. Some NPs increase Nox activity and can be used as antitumoral agents. The role of Nox 4 in vascular function is controversial; whereas some studies report a protective role against atherogenesis, others show the contrary. Certain NPs can be used as NO donors to reverse endothelial dysfunction. Some NPs exert SOD, catalase, oxidase, phosphatase, and peroxidase-mimetic activities.

Due to the abovementioned aspects, targeting the overproduction of H_2_O_2_ with nanoparticles could have beneficial effects on oxidative stress in cardiovascular diseases. In this regard, nanoparticles based on polyoxalate have been demonstrated to limit the effects of H_2_O_2_ on ischemia/reperfusion injury and have shown antioxidative and anti-inflammatory effects [187]. The use of nanoparticles as carriers has been championed widely [188]. In line with this, researchers have designed SOD1-carrying nanoparticles that have been shown to improve postmyocardial infarction cardiac function [189]. Although the role of nanoparticles as carriers is of undoubtable interest, this subject goes beyond the scope of the present review. Other reviews describe the main characteristics of nanocarriers with respect to the design new drug delivery systems [188, 190]. Some nanomaterials, such as nanoceria, have multienzyme mimetic activities. These nanoparticles are able to mimic SOD, catalase, oxidase, phosphatase, and peroxidase. Moreover, nanoceria can scavenge hydroxyl radicals and nitric oxide radicals [191]. In light of all of this, cerium nanoparticles exhibit great potential to treat diseases related to oxidative stress, as the majority of nanomaterials scavenge only a single type of RONS. In addition, nanoceria could have an anti-inflammatory effect due to their ability to scavenge NO.  Figure 5 shows the main mechanisms of vascular oxidative stress and antioxidant systems and the effects of nanoparticles on them.  Table 1 summarizes the different types of nanoparticles and their effects.

## 4. Conclusion

The aim of the present review is to analyse the effects of nanoparticles on oxidative stress in the vascular system. The elevated levels of RONS in the vascular wall are related to cardiovascular disease and a decreased bioavailability of NO, which leads to endothelial dysfunction. Some nanoparticles are antioxidants and may improve the vascular dysfunction associated with hypertension, diabetes mellitus, or atherosclerosis. However, other nanoparticles have displayed toxicity, as well as proinflammatory and prooxidant effects in endothelial cells. This toxicity seems to depend on the type and size of the nanoparticle in question. Nanoceria are one of the most promising types of nanoparticles in terms of restoring the oxidative balance and endothelial function. However, very few studies have focused on the effects of nanoceria on vascular reactivity, and so further research is needed in order to clarify the mechanism of these nanoparticles when interacting with the vascular system. We can conclude that, even though nanoparticles have extensive potential therapeutic applications in medicine, more toxicity studies are vital to acquire a greater understanding of this fascinating and promising technology.

## Figures and Tables

**Table 1:** Nanoparticles and their biological effects.

References	Nanoparticle studied	Function	Cell type
Manickam et al. [112]	Iron oxide nanoparticles	Oxidant by Nox 4 overexpression	Myocardium from mice
Petty [117]	WO_3_-Pt nanoparticles	Oxidant. NADPH oxidase biomimetic	Tumor cells
Sun et al. [113]	Silver nanoparticles	Oxidant by increasing Nox 4 expression	Human umbilical vein endothelial cells
Ramirez-Lee et al. [131]	Silver nanoparticles	Increase of hypertension due to a decrease in NO levels	Myocardium from rats
T. Yu et al. [142]	PLGA nanoparticles	Carrier. Treatment for hypertension	Hypertensive rats
Le et al. [132]	PLGA nanoparticles	ROS scavenger at vascular level and endothelial protector	Human umbilical vascular endothelial cells
Reddy and Labhasetwar [63]	PLGA nanoparticles	SOD carrier	Rat focal cerebral ischemia/reperfusion injury
Hennig et al. [141]	PEGylated quantum dots	Carrier of angiotensin II	AT1R-expressing cells
C. Guo et al. [158]	Silica nanoparticles	Endothelial injury induced by mitochondrial dysfunction	Human endothelial cells
Farooq et al. [157]	Silica nanoparticles	Endothelial dysfunction induced by oxidative stress	Aorta from rat
D. Lee et al. [71]	PVAX	Antioxidant, anti-inflammatory, and antiapoptotic activity	Hind-limb and liver from an ischemia/reperfusion model in mice
Marrache and Dhar [80]	PLGA-b-PEG-TPP nanoparticles	Nanocarriers	Mitochondria-acting therapeutics
Marushima et al. [73]	RNP	Neuroprotective agent due to its ability to scavenge free radicals	Middle cerebral artery from rats with cerebral ischemia/reperfusion injury
Chonpathompikunlert et al. [74]	Redox-polymer nanotherapeutics	Treatment of the neurodegenerative diseases	Brain from SAMP8 mice
Ciofani et al. [60]	Nanoceria	SOD and catalase mimetic	PC12 neuronal-like cells
Estevez et al. [69]	Nanoceria	Reduction of oxidative and nitrosative damage after stroke	Mouse hippocampal brain slice model of ischemia
Hirst et al. [67]	Nanoceria	Anti-inflammatory and NO scavenger	Murine macrophages
Niu et al. [152]	Nanoceria	Antioxidant	Cultured rat H9c2 cardiomyocytes
Niu et al. [161]	Nanoceria	Antioxidant and anti-inflammatory	Murine myocardium
Gojova et al. [164]	Nanoceria	Inflammatory effect	Human aortic endothelial cells
Wingard et al. [162]	Nanoceria	Vascular dysfunction	Aorta from mice
Minarchick et al. [159]	Nanoceria	Vascular antioxidant	Arterioles from hypertensive rats
Minarchick et al. [163]	Nanoceria	Prooxidant. Microvascular dysfunction	Arteriola from rats
Kennedy et al. [165]	Iron oxide, yttrium oxide, cerium oxide, zinc oxide	Proinflammatory	Human vascular endothelial cell line
Park et al. [187]	Nanoparticles based on polyoxalate	Antioxidant and anti-inflammatory	Doxorubicin-treated mice heart
Seshadri et al. [189]	Polyketal particles	SOD carrier	Rat myocardium

WO_3_-Pt: platinum tungsten oxide; PLGA: copolymer poly (lactic-co-glycolic acid); SOD: superoxide dismutase; PEG: polyethylene glycol; PVAX: copolyoxalate containing vanillyl alcohol (VA); RNP: radical-containing-nanoparticles.
